# A systematic review of theoretical constructs in CDS literature

**DOI:** 10.1186/s12911-021-01465-2

**Published:** 2021-03-17

**Authors:** Siru Liu, Thomas J. Reese, Kensaku Kawamoto, Guilherme Del Fiol, Charlene Weir

**Affiliations:** Department of Biomedical Informatics, University of Utah, Salt Lake City, Utah USA

**Keywords:** Clinical decision support, Unified Theory of Acceptance and Use of Technology, Taxonomy

## Abstract

**Background:**

Studies that examine the adoption of clinical decision support (CDS) by healthcare providers have generally lacked a theoretical underpinning. The Unified Theory of Acceptance and Use of Technology (UTAUT) model may provide such a theory-based explanation; however, it is unknown if the model can be applied to the CDS literature.

**Objective:**

Our overall goal was to develop a taxonomy based on UTAUT constructs that could reliably characterize CDS interventions.

**Methods:**

We used a two-step process: (1) identified randomized controlled trials meeting comparative effectiveness criteria, e.g., evaluating the impact of CDS interventions with and without specific features or implementation strategies; (2) iteratively developed and validated a taxonomy for characterizing differential CDS features or implementation strategies using three raters.

**Results:**

Twenty-five studies with 48 comparison arms were identified. We applied three constructs from the UTAUT model and added motivational control to characterize CDS interventions. Inter-rater reliability was as follows for model constructs: performance expectancy (κ = 0.79), effort expectancy (κ = 0.85), social influence (κ = 0.71), and motivational control (κ = 0.87).

**Conclusion:**

We found that constructs from the UTAUT model and motivational control can reliably characterize features and associated implementation strategies. Our next step is to examine the quantitative relationships between constructs and CDS adoption.

## Background

Clinical decision support (CDS) implemented in the context of an electronic health record (EHR) can enhance patient health and healthcare quality [1, 2]. CDS has been defined as the provision of patient-specific knowledge, information, and recommendations to clinicians in order to support optimal healthcare decisions [3]. Unfortunately, of published CDS interventions, approximately half are not associated with statistically significant improvements to clinical outcomes [4]. One possible reason for this variation in outcomes is user adoption. To understand the features that impact adoption, and, in turn, successful patient outcomes, we need more research to investigate the psychological mechanisms that are key to these types of interventions.

Previous systematic reviews have identified features that might increase CDS adoption [5–9]. For example, some CDS features found to have a positive impact include automatic data gathering and presentation, triggering presentation during decision making, and the provision of actionable recommendations [5]. While these systematic reviews do provide insight regarding particular features that may help CDS adoption, they don’t directly address the theoretical mechanisms of action. As a result, generalizable knowledge is limited. Specifically, reviews on existing features lack theoretical grounding in terms of identifying the relationship between how interventions are operationalized and the causal mechanisms that are being manipulated. This issue has been discussed for many years. As Shekelle et al. stated in their RAND’s comprehensive review of health information technology (HIT) interventions:

“In summary, we identified no study or collection of studies, outside of those from a handful of HIT leaders, that would allow a reader to make a determination about the generalizable knowledge of the system’s reported benefit. This limitation in generalizable knowledge is not simply a matter of study design and internal validity. Even if further randomized, controlled trials are performed, the generalizability of the evidence would remain low unless additional systematic, comprehensive, and relevant descriptions and measurements are made regarding how the technology is utilized, the individuals using it, and the environment it is used in [8].”

The pathway from the CDS intervention to adoption behavior begins with the psychological mechanism that is the core functionality of the system, through user beliefs, goals, intentions, and finally behavior. The Unified Theory of Acceptance and Use of Technology (UTAUT) model is a validated integrative model of theories, which can explain more than 70% of the variance in behavioral intention and 50% of the variance in user behavior (N = 399) [9]. The UTAUT model was developed by mapping and integrating 32 constructs over 8 well-known adoption theories. Intentions were used because a long-standing body of research has demonstrated that intentions were highly correlated with actual behavior [9]. The UTAUT model has been well validated across many systems. In healthcare, several studies have used the UTAUT model to explain and predict the IT acceptance, such as EHRs and online reporting systems. This body of work provides a reasonable and solid basis for applying the model to the CDS context [10–12].

We propose to use the UTAUT constructs to explore the psychological characteristics of CDS interventions and to predict adoption. The literature on CDS contains very few direct tests or manipulations of specific attributes that directly test a psychological mechanism. For example, CDS content is assumed to be better adopted if the information provided is relevant. However, few studies directly compare low versus high relevant information to test that hypothesis. Our goal is to explore the feasibility of using the UTAUT model to estimate the underlying mechanisms that might be manipulated in a head-to-head comparison of two forms of a CDS intervention. Although the UTAUT constructs were not directly manipulated, we propose to estimate them from the descriptions of the intervention itself, not through individual subjective assessments. In other words, we characterized the manipulation itself using the UAUT taxonomy.

We sought to adapt the UTAUT model to actual features that were tested in randomized controlled trial (RCT) studies by comparing one form of the CDS with another form and categorizing the difference between two groups; we only used RCTs, so the effect found can be rigorously assumed to be due to the manipulated factor. Instead of comparing the usual care with CDS interventions, comparative effectiveness studies use at least two different forms of active interventions (not usual care) to identify what intervention works best to improve outcomes. This restriction on the study design could help us to get the most accurate evidence [11] and use actual usage to measure adoption.

The ***objectives*** for this work are to examine the theoretical mechanisms of features influencing providers’ CDS adoption and to develop a taxonomy to describe interventions based on the current status of head-to-head comparative randomized evidence. To illustrate usage of the taxonomy, we provide three cases where CDS features are described and coded. This study is expected to contribute in both the technology adoption and CDS domains.

## Methods

This measurement study largely focuses on a conceptual approach, proposed by Bailey, for developing taxonomies [13]. We used constructs grounded in theory from the UTAUT model and the CDS literature.

### UTAUT

The UTAUT model includes four core constructs: (1) performance expectancy; (2) effort expectancy; (3) social influence; and (4) facilitating conditions [13]. Some of these are easily identifiable in the method sections of research studies and some are less so.

***Performance expectancy*** refers to the user’s belief that the CDS intervention can improve work performance. It involves five sub-constructs: perceived usefulness, extrinsic motivation (reward), job-fit, relative advantage, and beliefs about expected outcome. Based on the UTAUT model, performance expectancy is the strongest predictor of user intentions [9]. CDS tools are designed to be useful, but few studies actually measure performance expectancy, nor do they directly compare systems with different levels of performance expectancy. In the context of CDS interventions, level of performance expectancy can be determined by using direct evidence from interviews, usability surveys, or evidence of user-centered design processes.

***Effort expectancy*** refers to the perceived ease of use, or perceived effort using the CDS intervention. Effort expectancy integrates constructs that include perceived ease of use, complexity, self-efficacy, and anxiety [9]. Effort expectancy can be estimated by the time burden or need for sustained attention. A CDS intervention that decreases the number of clicks or provides more information in one place would be expected to have lower effort. Effort expectancy significantly affects early behavioral intentions, and the effect fades over time as users gain skills in using the CDS [9].

***Social influence*** refers to the degree of social pressure or social expectations to use the CDS intervention. Social influence consists of two parts, leadership pressures and peer social influence [9]. Social influence is also related to subjective norms (internalized beliefs of others’ expectations), and perceived image of oneself by others. The level of social influence was determined by several factors, including tools to monitor usage, the provision of performance feedback, comparison of user’s behavior with others, and programs to monitor compliance.

***Facilitating conditions*** encompass the infrastructure (both technical and organizational) and the strategies used to support and implement the CDS intervention. Instead of affecting behavioral intentions (i.e., performance expectancy, effort expectancy, and social influence), facilitating conditions can predict actual usage directly [9]. For CDS interventions, we considered facilitating conditions to be estimated in four parts: (1) IT infrastructure or user customization, such as the use of tailoring text or highlighting important information; (2) support for users, such as the provision of technical assistance to address hardware and software issues; (3) training, such as offering educational programs regarding how to use the tool; and (4) other facilitating factors, such as incentives to decrease the cost of unnecessary tests.

***Motivational control*** is a construct that we included to account for human agency, a factor that many researchers believe to be understudied in the area of clinical IT interventions. In addition, clinicians often complain that current EHR systems limit their ability to choose and act with agency [14]. This construct is similar to the construct of autonomy, a core and ubiquitous drive of humans as identified in Self-Determination Theory [15], Bandura’s Social Cognitive Theory [16] as well as Csikszentmihalyi’s Intrinsic Motivational Theory [17]. Because autonomy is a basic characteristic of professional roles, it is highly relevant to a discussion of the features associated with CDS adoption and use by clinicians. Perceived threat to professional autonomy has been identified as a critical negative impact on physicians’ acceptance of healthcare IT [18–22]. Perceived control has a long history in motivational psychology. For example, it was added to the Theory of Planned Behavior [23] to improve the predictive power of the Theory of Reasoned Action [24]. Validation studies of UTAUT addressed control construct by separating the validation into those studies where users could choose to the system versus those studies where users could not choose.

### Data sources

We used an iterative process to develop the taxonomy. First, we selected RCTs from multiple literature reviews related to CDS adoption features published after 2000 [6, 25–27]. We included only RCTs with a direct comparison of features that may affect CDS adoption. This restriction on the study design would improve the accuracy of the taxonomy by using the highest level of evidence [11]. The completed inclusion criteria were: (1) RCTs; (2) comparison of two forms of a CDS tool (the CDS tool with and without additional features); (3) providers received the CDS intervention; (4) examined CDS is integrated into the EHR; (5) all types of CDS: alerts, reminders, order sets, dashboards, infobuttons, documentation templates, and shared decision making tools [3]; (6) automatic extraction and use of data from the EHR; and (7) CDS that was used in inpatient acute care hospitals and in outpatient primary and sub-specialty care clinics. Two authors (SL and TR) participated in study selection and a third author (CW) resolved discrepancies. We extracted study characteristics (e.g., study design, setting, country, clinical area, and CDS type), detailed information including screenshots about each CDS intervention, and any information relating to the constructs.

### Taxonomy development

The preliminary taxonomy was based on the description of the UTAUT model [9, 23, 28]. The coding protocol began initially with the UTAUT model, and followed with an inductive review within each construct through group-based discussion. The iterative process started with two reviewers (SL and TR) independently coding 2–3 studies. Each study arm was coded with constructs in the UTAUT model and motivational control. CDS features were mapped to three levels of intensity (i.e., *High, Medium, and Low*) within constructs except for facilitating conditions. In developing a measurement protocol for the construct of “facilitating conditions,” we found that they were usually listed as parallel implementation strategies, making it difficult to determine which strategy facilitated and certainly we did not usually have any measures of support. We also did not have any direct measures of perceived support. Based on this concern, we counted how many implementation strategies used in the study as the coding measurement protocol, but fully understanding this limitation. Hopefully in the future, researchers will do a more thorough job of reporting and testing different strategies. Facilitation is key to implementation based on guidelines developed by the Agency for Healthcare Policy and Research (AHCPR) and is a core component in several implementation theories such as Consolidated Framework for Implementation Research (CFIR) [29], Promoting Action on Research Implementation in Health Services (PHARIS) [30], etc. In addition, studies that examine user’s perception of support and the relationship of those perceptions to actual strategies have shown significant relationship. As a result, counting facilitating conditions is a reasonable proxy. Additionally, we compared the CDS intervention coding between study arms. The group (SL, TR, and CW) met and discussed coding discrepancies and revised the taxonomy with each set of 2–3 studies. This process was repeated until two sets of subsequent studies did not result in taxonomy changes and achieved high inter-rater reliability (Cohen’s κ > 0.80) [31]. An expert in social psychology theory (CW) discussed and adjudicated differences to help ensure the coding quality. To validate the taxonomy, a new set of studies was independently coded by two authors (SL and TR).

## Results

We identified 25 studies meeting the inclusion criteria. Within the included studies, 48 comparison arms were coded. Most studies used a cluster RCT design (88%). The mean number of sites, number of subjects, and study time (months) were 15.2, 16,150, and 14.7, respectively. Studies were conducted in the United States (18), the Netherlands (3), Canada (2), New Zealand (1), and the United Kingdom (1). Twenty-three studies (92%) were conducted in an outpatient setting and two in an inpatient setting. Practice settings included general practice, gynecology, and pediatrics. Table 1 provides characteristics of the CDS interventions.

**Table 1:** Characteristics of included CDS interventions

Characteristic	Percentage (%)
*Clinical domain *(*n = 25*)	
Management of chronic medical condition or preventive care	48
Pharmacotherapy	36
Laboratory test ordering	8
Diagnosis	4
Immunization	4
Management of psychiatric condition	4
*Clinical decision support type *(*n = 25*)	
Alert	60
Reminder	24
Order set	16
Dashboard	16
Infobutton	4

### Taxonomy results

After four iterations and 10 studies, we reached an acceptable agreement for each construct. The taxonomy was validated by coding an additional 15 studies. Table 2 provides the taxonomy validation agreement and the coding rules for each level within constructs. The coding schema had two parts for each construct: (1) the operational definition of the construct in terms of CDS context and (2) rules to assess levels of intensity using an ordinal scale in order to determine the degree of performance expectancy, effort expectancy, social influence, or motivational control, as well as to extract facilitating conditions. The average inter-rater reliability was κ = 0.81, which shows high levels of agreement across constructs [32].

**Table 2:** Coding criteria in the taxonomy and inter-rater reliability

Performance expectancy (IRR: 0.79)	High	(Researchers conducted interview(s) before designing the CDS tool **OR** Researchers performed usability testing with at least five users and more than 75% of participants agreed the CDS tool was useful **OR** Clinicians agreed that CDS tool provided patient-specific recommendation.) **AND** The CDS tool was context sensitive (e.g., CDS triggered by workflow events and CDS targeted to the right providers)
	Medium	(Researchers performed usability testing with at least five users and 50%-75% of participants agreed the CDS tool was useful **OR** The CDS tool was context sensitive.) **AND** Local users were involved in developing the CDS tool [33]
	Low	Researchers performed usability testing and less than 50% participants agreed the CDS tool was useful **OR** Local users were involved in developing the CDS tool [33] **OR** The CDS tool was context sensitive
	Unknown	Not enough relevant information mentioned in the article
Effort expectancy (IRR: 0.85)	High	The CDS tool interrupted the workflow **OR** The CDS tool required a lot of effort to use (e.g., providing the dashboard, requesting documentation of reason for any non-compliance [33], and at least some manual input of values [34]) **OR** The CDS tool required clinician initiative to use [33] **OR** The estimated user interaction time was greater than 3 s **OR** 75% + of users reported that the CDS tool required high effort to use
	Medium	The CDS tool interrupted the workflow and required moderate cognitive load (e.g., users needed to click one or few buttons to accept or dismiss, had a pop-up window to display the message) **OR** The estimated user interaction time was 1–3 s **OR** 50–74% of users reported that the CDS tool required high effort to use
	Low	The CDS tool was quick and required less than 1 s to use. Users did not need to find information somewhere else **OR** 50% + of users reported that the CDS tool was easy to use
	Unknown	Not enough relevant information mentioned in the article
Social influence (IRR: 0.71)	High	Peer pressure existed during the implementation (e.g., rated user’s performance, patient was encouraged to be involved in the decision) **OR** Participants knew that they were being monitored on an individual basis (e.g., written justification was visible in the EHR [35], 1-to-1 feedback) **OR** CDS was a part of a larger program implementation (e.g., falls program) **OR** CDS was part of a regulatory measure (e.g., electronic clinical quality improvement)
	Medium	Researchers provided an education program for all of the users **OR** Users received a communication from the supervisor about use of the CDS tool **OR** CDS was targeted toward more than one provider of practice group
	Low	No tracking or no monitoring existed in the program **OR** Researchers provided an education program for some users **OR** Researchers did not provide an education program **OR** CDS was targeted toward patients
	Unknown	Not enough relevant information mentioned in the article
Facilitating conditions		A. IT infrastructure or user customization (e.g., tailored the timing and frequency of prompts, tailored text, highlighted text, links to supporting information, make justification publicly visible, and interactive dashboard) B. IT support for users (e.g., technical assistance to address hardware and software issues) C. Training (e.g., the specialized instruction concerning the system provided) D. Other facilitating factors (e.g., economics decrease the cost for the unnecessary tests)
Motivational control (IRR: 0.87)	High	Users could simply ignore the CDS tool, (e.g. low control on their behavior, passive alerts, and a changeable alert threshold)
	Medium	Users had to respond to the CDS tool but involved low effort, (e.g. pop up with easy exit out)
	Low	Users were forced to use the CDS tool with at least medium effort (e.g., pop up with text input, interrupt the workflow, and cannot ignore it)
	Unknown	Not enough relevant information mentioned in the article

IRR, inter-rater reliability; CDS, clinical decision support; IT, information technology

### Case examples

We selected three cases to illustrate how the taxonomy and coding schema (Table 2) were used to characterize the CDS interventions. We chose these three cases to illustrate the breath of constructs impacted. The first case illustrates the nature of facilitating conditions; the second case illustrates effort expectancy and motivational control; and the last case illustrates how all of the constructs except performance expectancy can be affected. These examples demonstrate how additional features can interact together, and how changing one construct level may cause other constructs to change.

The first case aimed to test the effect of adding more contextual information into a CDS tool in the outpatient setting [36] (Table 3). The control arm was a drug-drug interaction alert related to hyperkalemia. The intervention was the same alert but with added specific patient laboratory data. Applying our model to compare these two arms, we found that the additional feature, presenting lab data, adds only one facilitating condition and the other four constructs were unchanged. In both arms, performance expectancy was low because researchers did not report usability testing or interviews. In addition, clinicians in the discussion group determined that provided information was not helpful for users. The drug-drug interaction alert was quick and required less than one second to use. Therefore, effort expectancy was low, and motivational control was medium in both arms because users had to respond to the alert. No education program or compliance tracking system was provided, leading to low social influence in this experiment.

**Table 3:** Coding of the first case—drug-drug interaction alert (Duke, 2013 [36])

Performance expectancy	Effort expectancy	Social influence	Facilitating conditions	Motivational control
*Control: Drug-drug interaction alert*				
Low Trigger by drug-drug interactions associated with hyperkalemia	Low The interaction time less than one second	Low No education or tracking program	1) provide explanation links	Medium Need user response; low efforts
*Intervention: Drug-drug interaction alert tailored with specific patient laboratory data (most recent potassium and creatinine levels)*				
Low Show relevant lab values with the alert	Low The interaction time less than one second	Low No education or tracking program	1) provide explanation links; 2) tailored alert text	Medium Need user response; low efforts
*Change between study arms*				
−	−	−	+ 1	−

In this case, we explain why we classified the provision of laboratory data as having a low level of performance expectancy. While a CDS with lab-specific data might increase usefulness, in developing the taxonomy, we found that most of the current CDS research has recognized the importance of introducing more patient-specific data and have implemented such CDS in clinical practice. Therefore, the more important information for determining the level of performance expectancy is whether or not usability testing was conducted. It ensures that the additional information provided meets user needs and avoids information overload. It is also important to note that CDS should not simply list patient-specific information. Rather, the CDS should provide a recommendation based on the patient-specific information in order to be useful. In this study, the CDS would have been more useful from the clinician's perspective if it had provided specific recommendations based on the different values of the laboratory data, rather than just displaying values.

The second case shows a dynamic relationship between effort expectancy and motivational control (Table 4). Scheepers-Hoeks et al*.* compared CDS on demand with a pop-up alert for medications in the intensive care setting [37]. This comparison decreased effort expectancy from high to low and motivational control from high to medium. In the CDS-on-demand arm, effort expectancy was high, because providers needed to remember to activate the CDS in the EHR. With the pop-up alert, information was shown automatically, which decreased effort expectancy. Motivational control was high in the control arm, because providers could ignore the CDS tool. In the arm of pop-up alerts, the motivational control was medium, because the pop-up alert required users to respond with low effort. The CDS tools in both arms were locally customized and provided patient-specific recommendations, which supported high performance expectancy. Social influence was medium in both arms because of the educational program provided to all users, which would convey social or work obligations. After changing an on-demand CDS to a pop-up alert, providers could use the tool with less effort; however, they lost control to some degree. This case also shows the necessity of adding the motivational control in the model to characterize CDS interventions comprehensively. Changing a CDS on demand to a pop-up alert would decrease the effort to use the tool but also affect user autonomy and perceived control.

**Table 4:** Coding of the second case—medication CDS in the ICU (Scheepers-Hoeks, 2013 [37])

Performance expectancy	Effort expectancy	Social influence	Facilitating conditions	Motivational control
*Control: CDS on demand as an EHR section*				
High Customized, patient-specific recommendation	High Users have to remember to use the CDS	Medium Education program	(1) education; (2) tailored alert text	High Simply ignore
*Intervention: Pop-up alerts*				
High Customized, patient-specific recommendation	Low Pop up automatically	Medium Education program	(1) education; (2) tailored alert text	Medium Pop-up alerts, easy exit out
*Change between study arms*				
−	High → Low	−	−	High → Medium

In the third case, Meeker et al. assessed the effect of three behavioral interventions to reduce inappropriate antibiotic prescribing [35] (Table 5). Suggested alternatives alone were compared to the combination of suggested alternatives with accountable justification and peer comparison. Providers in the first arm received order sets to notify the user of suggested treatments. In the second arm, a prompt to enter a justification for overriding the alert was added to the suggested alternatives. Providers also received email feedback that provided their antibiotic prescribing rates and the lowest inappropriate prescribing rates from peers. This intervention changed effort expectancy and social influence from medium to high, motivational control from medium to low, and added two facilitating conditions. Effort expectancy was increased because of the need for manual input. Two aspects caused social influence to increase: one was the publicly visible justification, and another was peer pressure. Motivational control was decreased because providers were required to respond with high effort. The two facilitating conditions included feedback on inappropriate prescriptions and a requirement for public justification. This case emphasizes the importance to apply the UTAUT model with the motivational control to design or optimize CDS. In this study, researchers added two more behavioral intentions, however, the change between study arms didn’t have an impact on the performance expectancy.

**Table 5:** Coding of the third case—CDS to reduce inappropriate antibiotic prescribing (Meeker, 2016 [35])

Performance Expectancy	Effort Expectancy	Social Influence	Facilitating Conditions	Motivational Control
*Control: Order sets to notify suggested treatments*				
Low Context sensitive	Medium Interrupt workflow, moderate cognitive load	Medium Train all users	(1) education; (2) drop-down list; (3) link to order sets; (4) link to documentation templates	Medium Response with low efforts
*Intervention: Order sets to notify suggested treatments, accountable justification, and peer comparison*				
Low Context sensitive	High Write, high cognitive load	High Public written justification, peer pressure	(1) education; (2) drop-down list; (3) link to order sets; (4) link to documentation templates; (5) feedback on inappropriate prescriptions; (6) public justification	Low Pop-up with text input
*Change between study arms*				
−	Medium → High	Medium → High	+ 2	Medium → Low

## Discussion

We found that a taxonomy based on the UTAUT model and motivational control could not only be used to reliably characterize CDS interventions in the primary literature, but also to characterize the effect and an estimated mechanism of action. Moreover, adding the motivational control construct was needed to address the variation in how CDS interventions impact user autonomy. In validating the taxonomy, we achieved substantial to near perfect agreement when classifying CDS features in model constructs. The UTAUT model constructs and motivational control can be applied systematically and reliably to the CDS domain while providing generalizable knowledge. A future analysis of the larger CDS domain will allow us to evaluate the importance and predictive value of these theoretical constructs on outcomes. This work should encourage researchers to formally test specific hypotheses and theoretical mechanisms to understand the impact of CDS features.

Our modification of the UTAUT model, by adding motivation control, is congruent with other research in this area. While the UTAUT model was developed to robustly explain adoption and use of IT, researchers have been encouraged to modify the model based on unique characteristics of users and technology [9]. For instance, Alaiad and Zhou added a *trust* construct when developing a theoretical model of patient acceptance of a health care robot [38]. Hoque et al. added *technology anxiety* and *resistance to change* to examine factors that affect the adoption of mHealth by the elderly [39]. Another study adapted UTAUT by adding *compatibility* and *self-efficacy* constructs to explain acceptance and satisfaction of nurses using an EHR [40]. Finally, Chang et al. predicted providers’ intention to use an online reporting system in 2012 by combining the UTAUT model with the value of perceived consequence, which can explain users’ subjective values when using the system [10]. In the IT domain with the context of EHRs, modification of the UTAUT model was needed to account for the variable levels of autonomy offered by IT applications.

Our approach differs from other studies in four aspects. First, we operationalized the UTAUT constructs specifically for the CDS context using a formal approach of developing a reliable taxonomy. Second, we operationalized the constructs in terms of CDS features and not subjective judgments. This variation in how constructs are operationalized is not unusual in the experimental psychology literature, as most experimental studies directly test the casual impact of psychological mechanisms through explicit manipulations designed to create that effect. Of course, future work should both manipulate variables and measure their psychological impact subjectively and on behavior. Third, we applied the UTAUT model to investigate features from user psychological perspective. Comparing with the socio-technical system models (e.g. SEPIS [41]), our method targeted on individual behavior and their acceptance to a technology. Socio-technical system models often provide components and identify interactions between user, technology, and organization to better design or assess a whole system. Fourth, we selected RCTs in comparative effectiveness studies to develop and validate the taxonomy. This study type compares two CDS interventions with and without some features, which could provide more accurate evidence in evaluating the features’ impacts on CDS success [42].

Limitations of this study should be noted. First, in many respects, the accuracy of coding results is influenced by the reporting quality of the selected RCTs. For example, if a study did not report conducting user interviews in the selected study or a previous study, then we assumed they did not conduct interviews. Second, our study used the CDS literature to develop the taxonomy instead of conducting surveys. Publication bias is likely prevalent in the CDS literature. Third, when researchers use our findings to guide CDS design and implementation, they need to be cautious and carefully examine the overall usefulness of the system carefully. For example, when evaluating performance expectancy, we had two clinicians to determine whether the CDS tool provided patient-specific recommendations. The pre-implementation testing (e.g. usability testing) should not be ignored. Finally, constructs in the UTAUT model and motivational control have not been empirically validated in their ability to predict adoption behavior and patient outcomes. Our next step is to conduct meta-regression to explore how constructs relate to the effectiveness of CDS interventions.

## Conclusion

This study demonstrated the feasibility of characterizing CDS interventions in terms of a behavioral theory. We successfully developed a reliable coding schema using a validated behavioral theory model for HIT. The provided taxonomy can be used to create generalizable knowledge from the primary literature and guide future CDS development, implementation, and evaluation to maximize the chance of user acceptance and CDS adoption.

## Data Availability

All data generated or analyzed during this study are included in this published article.

## Abbreviations

CDS: Clinical decision support
HIT: Health information technology
IT: Information technology
UTAUT: Unified Theory of Acceptance and Use of Technology
κ: Kappa
EHR: Electronic health record
RCTs: Randomized controlled trials
IRR: Inter-rater reliability
AHCPR: Agency for Healthcare Policy and Research
CFIR: Consolidated Framework for Implementation Research
PHARIS: Promoting Action on Research Implementation in Health Services
